# Totally Laparoscopic Gastrectomy for Gastric Cancer Associated with Recklinghausen's Disease

**DOI:** 10.1155/2010/682401

**Published:** 2010-06-23

**Authors:** Yoshihisa Sakaguchi, Osamu Ikeda, Kippei Ohgaki, Eiji Oki, Yoshiki Chinen, Yasuo Sakamoto, Kazuhito Minami, Yasushi Toh, Takeshi Okamura

**Affiliations:** Department of Gastroenterological Surgery, National Kyushu Cancer Center, 3-1-1 Notame, Minami-ku, Fukuoka 811-1395, Japan

## Abstract

This paper documents the first case of gastric cancer associated with Recklinghausen's disease, which was successfully treated by a totally laparoscopic operation. A 67-year-old woman with Recklinghausen's disease was referred to this department to undergo surgical treatment for early gastric cancer. The physical examination showed multiple cutaneous neurofibromas throughout the body surface, which made an upper abdominal incision impossible. Laparoscopic surgery requiring only small incisions was well indicated, and a totally laparoscopic distal gastrectomy with lymph node dissection was performed. Billroth I reconstruction was done intra-abdominally using a delta-shaped anastomosis. The patient followed a satisfactory postoperative course with no complications. Since the totally laparoscopic gastrectomy has many advantages over open surgery, it should therefore be preferentially used as a less invasive treatment in the field of gastric cancer.

## 1. Introduction

Recklinghausen's disease is an autosomal dominant disorder characterized by cutaneous hyperpigmentation and multiple neurofibromas [1]. This disease shows a high incidence of malignant tumors, but most of those are neural tumors [2, 3]. There have been a few reports of gastrointestinal cancer with Recklinghausen's disease [4–6], and gastric cancer is rare as well [7]. In the surgical treatment of these cases, the skin incision may be limited by the presence of cutaneous neurofibromas. Recently, laparoscopic surgery has been accepted as the preferred treatment of gastrointestinal cancer [8]. The primary advantage of this type of surgery is that it is less invasive, and the procedure requires only small skin incisions. Therefore, a laparoscopic operation would be useful for patients with numerous cutaneous neurofibromas. This report describes the first case of gastric cancer associated with Recklinghausen's disease, which was treated by a totally laparoscopic operation.

## 2. Case Report

A 67-year-old woman with a history of Recklinghausen's disease was referred to this department for the surgical treatment of gastric cancer. The patient had been diagnosed to have gastric cancer at a previous hospital. Gastrofiberscopy showed a small elevated lesion (type IIa) in the lesser curvature of the middle portion of the stomach, and the histological diagnosis was well-differentiated tubular adenocarcinoma. An endoscopic mucosal resection was performed, and the lesion was thus removed completely. However, a follow-up endoscopic examination revealed another depressed lesion (type IIc) near the scar of the mucosal resection, seven months later. A biopsy revealed a histological diagnosis of moderately differentiated tubular adenocarcinoma. Clinical finding of submucosal invasion of the tumor indicated that surgery was necessary. 

On admission, the patient had no complaints. Physical examination showed multiple skin tumors (cutaneous neurofibromas) on all of the body surfaces, including the abdominal wall. The results of the laboratory tests were unremarkable, except for a white blood cell count of 3000/mm^3^. Tumor markers, including CEA and CA19-9, were within normal limits. Abdominal ultrasonography and computed tomography revealed no signs of metastasis. 

**Figure 1:**
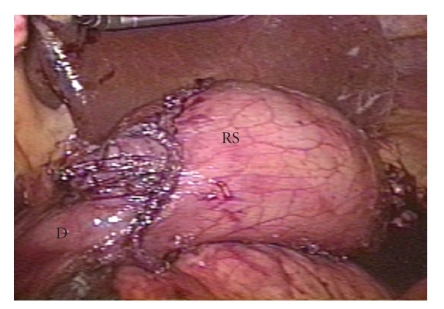
Laparoscopic image showing the intra-abdominal Billroth I reconstruction. The gastroduodenostomy was completed using only linear staplers. RS: remnant stomach; D: duodenum.

**Figure 2:**
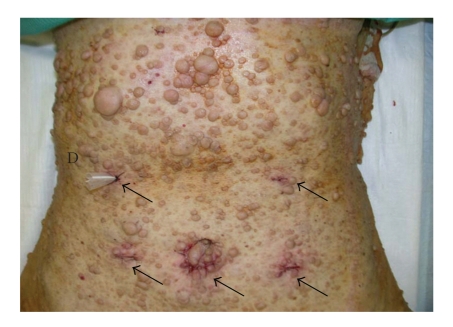
Many cutaneous neurofibromas crowded on the abdomen, especially epigastric area. All incisions (arrows) were small, and the operation was finished without injuring the skin tumors. D: drain.

 A totally laparoscopic distal gastrectomy with lymph node dissection (D2) was performed. The procedures were done according to the method described by Kanaya et al. [9]. To perform pneumoperitoneum and operate the devices, five trocars were inserted into the peritoneal cavity, while carefully avoiding the skin tumors. Following the gastrectomy, the lymphadenectomy, and the reconstruction were all done intra-abdominally. After mobilization of the gastroduodenum and a sufficient lymphadenectomy, the duodenum and the stomach were transected using endoscopic linear staplers (ETS Flex 45-3.5, Ethicon Endo-Surgery, Cincinnati, OH, USA). The resected specimen was removed out through the extended umbilical incision using a plastic tissue bag. A Billroth I gastroduodenostomy was performed by making delta-shaped anastomosis using the endoscopic linear staplers (Figure 1). The operation was completed uneventfully (Figure 2). The number of dissected lymph nodes was 38. The duration of the operation was 260 minutes and the blood loss was 140 g. The patient followed a satisfactory postoperative course with no complications, and was discharged in excellent condition 10 days after the operation.

**Figure 3:**
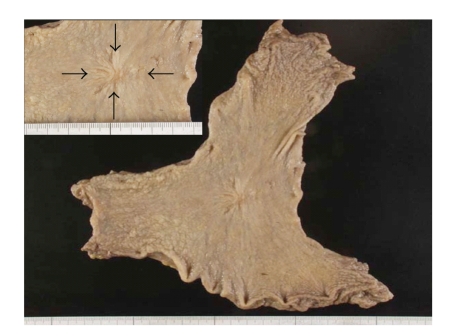
A macroscopic examination of the resected specimen showed an irregular depressed lesion (type IIc), measuring 3.0 × 2.5 cm (arrows), in the lesser curvature of the middle portion of the stomach.

 A histological examination showed that the lesion was slightly depressed (type IIc) and measured 3.0 × 2.5 cm (Figure 3). The cancer was a moderately differentiated tubular adenocarcinoma, partially invading to the submucosa of the gastric wall without vessel invasion. None of the dissected lymph nodes showed any metastasis. 

## 3. Discussion

Recklinghausen's disease (peripheral neurofibromatosis) is an autosomal dominant disorder characterized by cutaneous hyperpigmentation (café au lait spot) and multiple neurofibromas [1]. It is associated with a high incidence of neural tumors, such as optic glioma, glioblastoma, and meningioma [2]. Some reports have also demonstrated an association of nonepithelial tumors in gastrointestinal tract [3]. In contrast, there have been a few reports of primary adenocarcinoma of the gastrointestinal tract, such as the stomach, small intestine, or colon [5–7]. The present study reports a rare case, which was associated with gastric cancer without neural tumors other than cutaneous neurofibromas. Because the relationship between Recklinghausen's disease and gastrointestinal cancer is still not fully understood, careful surveillance of gastrointestinal tract should be performed when considering the association of malignant tumors.

In the surgical treatment for the patients with Recklinghausen's disease, there is a possibility that the cutaneous neurofibromas may disturb the surgical process. When skin tumors exist in the area of a proposed incision for removing lesion, the site of the incision must be shifted. As a result, such a modification may hinder the surgical procedures. In addition, these patients tend to be self-conscious about their appearance and show more concern about surgical scars, than ordinary patients do. The present patient had many crowded neurofibromas in the epigastric area, and it would have been difficult to make an upper abdominal incision for the gastrectomy. Moreover, she was worried about the additional damage to her appearance, that would result from conventional surgical treatment. From these reasons, laparoscopic surgery requiring only small incisions was performed to resect the gastric cancer. 

Laparoscopic surgery is a less invasive treatment with faster recovery time, less pain, and less scaring than conventional open surgery. It has become popular in many fields of surgery [8]. In the treatment of gastric cancer, laparoscopic surgery has been adopted since 1991. In 1994, laparoscopy-assisted distal gastrectomy (LADG) with lymph node dissection was first applied by Kitano et al. [10]. Since the safety and surgical quality of LADG have been proved to be equivalent to open gastrectomy, LADG has been accepted as a less invasive treatment for early gastric cancer [11–13]. Recently, in addition to the short-term advantages of the procedure, satisfactory long-term outcomes have been demonstrated in multiple studies [14–16].

LADG usually requires a small laparotomy in the upper abdomen to remove the resected specimen from the abdominal cavity and to do the reconstruction. However, advances in laparoscopic surgical techniques have made it possible to now perform all of the necessary surgical procedures, including an intra-abdominal reconstruction [17, 18]. In addition, the development of endoscopic stapling devices has made intra-abdominal anastomosis easier and safer. The intra-abdominal anastomosis is advantageous because it is not influenced by the patient's physique or the location of disease. A Billroth I gastroduodenostomy is a standard reconstruction method used after a distal gastrectomy in Japan, and the extra-abdominal approach has usually been applied for the Billroth I anastomosis in LADG [12, 14]. In a totally laparoscopic distal gastrectomy (TLDG), the Billroth I gastroduodenostomy can also be performed intra-abdominally. One of the effective techniques is to make a delta-shaped anastomosis, which was first demonstrated by Kanaya et al. [9]. The procedure is derived from the application of the functional end-to-end technique to the anastomosis between the remnant stomach and the duodenum, and therefore requires only linear staplers. The present report demonstrates the efficacy of TLDG for a patient with Recklinghausen's disease. In addition, the usefulness can be applied to ordinary patients with gastric cancer. 

## 4. Conclusion

Since TLDG makes the best use of advantages of laparoscopic surgery, comparing to LADG, TLDG should therefore become widespread in the surgical treatment of gastric cancer.
